# Pachymic acid alleviates circadian rhythm disorders in high-fat diet-induced obesity mice via the sphingolipid pathway

**DOI:** 10.1371/journal.pone.0352604

**Published:** 2026-07-01

**Authors:** Hong Yang, Yingxiu Mei, Jilan Chen, Jiawei Chen, Jiushuang Zhu, Lu Zhang, Yanju Gong, Gan Luo, Weijun Ding

**Affiliations:** 1 School of Medical and Life Sciences, Chengdu University of Traditional Chinese Medicine, Chengdu, China; 2 School of Basic Medical Sciences, Chengdu University of Traditional Chinese Medicine, Chengdu, China; 3 Department of Orthopedics, Chengdu Integrated Traditional Chinese Medicine & Western Medicine Hospital/Chengdu First People’s Hospital, Chengdu, China; Wuhan Polytechnic University, CHINA

## Abstract

**Background:**

Obesity caused by a high-fat diet (HFD) is known to disrupt metabolic homeostasis and circadian rhythms. Pachymic acid (PA), a bioactive triterpenoid, exhibits anti-inflammatory, antihyperglycemic, antihyperlipidemic, and sedative-hypnotic properties, though its role in circadian regulation remains unexplored.

**Methods:**

We assessed PA’s impact on metabolic dysfunction (glucose/lipid profiles), systemic inflammation using biochemical assays, ELISA, Oil Red O staining. Circadian parameters were evaluated via 24-h serum melatonin and core body temperature. Hepatic circadian gene oscillations and mechanistic pathways were analyzed through time-series RNA sequencing, bioinformatics, qPCR, and Western blotting.

**Results:**

PA intervention attenuated obesity-related phenotypes, including reduced body weight, improved glucose/lipid metabolism, and restored physiological rhythms of melatonin and body temperature. And hepatic gene oscillation patterns were realigned to circadian synchrony. Mechanistically, PA ameliorated liver inflammation by modulating the sphingolipid pathway, specifically via *S1PR4/TRAF2* signaling.

**Conclusions:**

Our findings illustrate PA’s role in mitigating metabolic and circadian disruptions in obesity, highlighting the sphingolipid pathway as a tissue-specific target for circadian modulation. This study provides novel insights into therapeutic strategies for obesity-associated circadian disorders.

## Introduction

Obesity has emerged as a critical global health crisis, significantly contributing to the development of multiple chronic diseases such as cardiovascular disorders, type 2 diabetes mellitus, obstructive sleep apnea, various malignancies, and degenerative joint diseases like osteoarthritis [1]. Owing to a sharp increase in incidence over recent decades, greater attention is now being given to prevention, treatment, and strategies for mitigating associated health risks [2,3]. Overnutrition, especially a high-fat diet (HFD), is the main cause of obesity and metabolic disorders [4,5].

Mounting evidence indicates that circadian rhythms and dietary patterns are intricately interconnected in a bidirectional relationship. On one hand, nutrient intake serves as a powerful zeitgeber time for peripheral clocks, with the composition and timing of diet critically shaping circadian oscillations [6]. High-fat diets, in particular, profoundly disrupt circadian homeostasis through multiple pathways: they induce hormonal imbalances (e.g., leptin and ghrelin resistance) [7], directly alter clock gene expression in peripheral tissues including the liver, adipose tissue, and skeletal muscle [8], and trigger chronic low-grade inflammation in metabolic tissues (liver, hypothalamus, and adipose tissue), which further impairs the circadian clock’s sensitivity to metabolic cues [9]. Moreover, HFD consumption alters the rhythmic expression of core clock genes such as BMAL1, CLOCK, PER, and CRY, leading to desynchronization of downstream metabolic pathways [10].

Conversely, disrupted circadian rhythms reciprocally exacerbate obesity and metabolic dysfunction. Clock gene deficiencies and circadian misalignment promote hyperphagia, reduced energy expenditure, impaired lipid metabolism, and enhanced adipogenesis [11]. Specifically, circadian disruption alters glucose homeostasis by blunting insulin sensitivity and delaying glucose clearance, while simultaneously promoting lipogenesis through regulatory element-binding protein (SREBP) [12]. Furthermore, clock-driven dysregulation of appetite-regulating hormones (e.g., suppressed leptin and elevated ghrelin) promotes excessive caloric intake and weight gain [13]. These findings underscore a vicious cycle: dietary-induced circadian disruption aggravates metabolic imbalance, which in turn further destabilizes circadian rhythmicity, creating a self-reinforcing loop that accelerates obesity progression. Time-restricted feeding (TRF) or calorie-restricted feeding (CRF) has been demonstrated to have antiobesity effects and the ability to modulate circadian rhythm disorders [14]. However, both TRF and CRF can be difficult to maintain long-term because of social situations, busy schedules, travel, and hunger, which can hinder consistent adherence [15]. A HFD disrupts circadian rhythms via multiple mechanisms: hormonal imbalance (leptin and ghrelin) [7], directly impacting clock gene expression in peripheral tissues [8], chronic low-grade inflammation affecting various tissues (liver, hypothalamus, and adipose tissue) which altering lipid metabolism, glucose homeostasis, and insulin sensitivity, thereby disrupting the circadian clock’s sensitivity to metabolism [9]. It helps identify regulatory mechanisms and allows the study of how disruptions to circadian rhythms can affect the body.

*Poria cocos* is an established Chinese herb as well as a health food. Pachymic acid (PA), a principal triterpenoid bioactive component, is isolated from *Poria cocos*. Recent studies have revealed that PA possesses potent anti-inflammatory [16], antihyperglycemic [17], hypolipidemic [18], and sedative-hypnotic [19] effects. Research has revealed the effects of PA on metabolic diseases, including nonalcoholic fatty liver disease and obesity [17,20]. Emerging evidence indicates that circadian clock disruption manifests during the initial phase of obesity, while mild inflammatory modulation and metabolic interventions can concurrently restore rhythmicity and ameliorate metabolic dysregulation [21].

Given the pivotal role of circadian clock dysfunction in obesity pathogenesis, we hypothesize that PA may exert its antiobesity effects, at least in part, by restoring circadian rhythmicity that is disrupted by HFD consumption. Therefore, understanding how PA modulates the molecular clock is essential for elucidating its therapeutic mechanism beyond its known metabolic benefits. However, whether and how PA affects the circadian clock system in the context of obesity remains unknown. This study evaluates the impact of PA on obesity-related circadian dysregulation using a HFD mouse model. Time series RNA-seq [22] was employed to systematically characterize how PA restores circadian rhythmicity at the transcriptome level, identifying core clock genes and downstream metabolic pathways that mediate its therapeutic effects.

## Materials and methods

### Ethics approval

This study was approved by the Committee Chengdu University of Traditional Chinese Medicine laboratory Animal Welfare Ethics Committee (No. 2024107). At the experimental endpoint, all animals were humanely euthanized via deep anesthesia induced by pentobarbital (100 mg/kg, i.p.), followed by cervical dislocation to ensure minimal distress. The mouse experiments were carried out in accordance with the ARRIVE guidelines.

### Preparation of chemicals

Both pachymic acid (purity > 98%) and melatonin (purity > 98%) were obtained from Ruifensi Co., Ltd. (Chengdu, China). Pachymic acid was dissolved in water containing 5% PEG300, the final concentration 1 mg/ml. Additionally, melatonin was dissolved in 5% PEG300 aqueous solution at a final concentration of 1 mg/ml.

### Animals and experimental design

**Fig 1:**
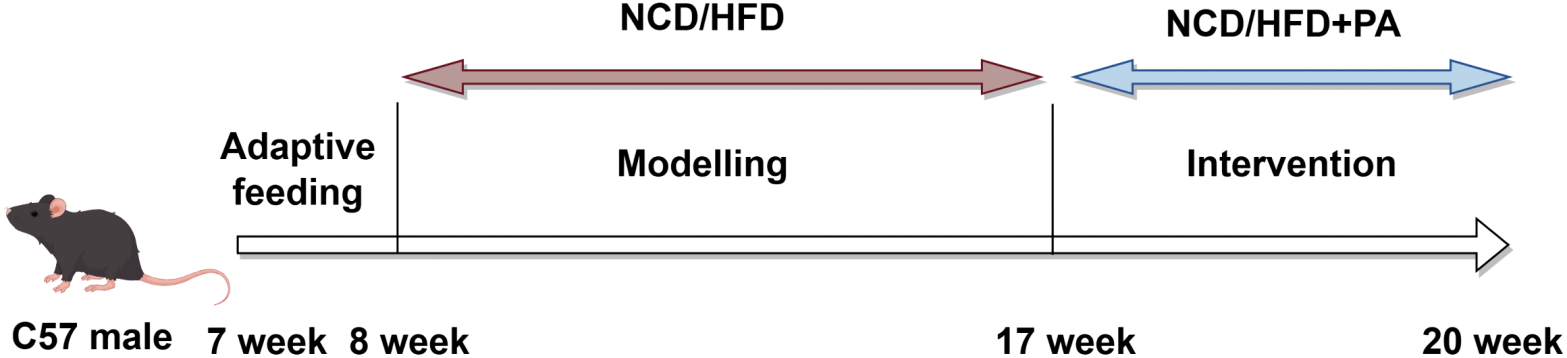
Flowchart of the experimental design. NCD: low-fat diet group; HFD: HFD-fed group; PA: HFD-fed with PA intervention group, And the numbers refer to age in weeks.

7-week-old, C57BL/6J wild-type male mice were obtained from Spfbiotech Co., Ltd. (Beijing, China; license number: SCXK(J)2019–0010). All mice were housed under a 12:12 hours light-dark cycle meaning light below 150 lux/ dark in 0 lux with Specific Pathogen-Free (SPF) conditions at a constant 23 ± 2°C and controlled humidity. The Zeitgeber time (ZT) [23] was used, with ZT0 defined as the time of lights on (8 AM), then ZT12 as the time of lights off (8 PM). In this study, all mice were housed under standard conditions with ad libitum access to chow and drinking water. After completing a 7-day environmental adaptation phase, eight-week-old mice were randomly allocated into two dietary intervention groups for a 9-week period: one cohort received a high-fat diet (HFD, 60% fat-derived calories, D12492, Research Diets, USA), while the control group was maintained on an isocaloric low-fat diet (LFD, 10% fat-derived calories, D12450J, Research Diets, USA). The obesity mice (HFD-fed mice weighing at least 20% higher than the average LFD-fed mice) were randomly stratified into five sub-groups: the HFD, high-dose PA (PAH, 10 mg/kg), medium-dose PA (PAM, 5 mg/kg), low-dose PA (PAL, 1 mg/kg) or melatonin (MT, 10 mg/kg). The low-fat diet group served as the normal control diet (NCD). Mice were injected intraperitoneally once daily for three weeks at 8:00–9:00 AM. To minimize the masking effect of feeding on circadian gene expression, mice were fasted for 8 hours prior to tissue collection at each designated ZT. Specifically, food was removed 8 hours before the scheduled sacrifice time for each time point group (e.g., fasting for the ZT8 group started at ZT0, and for the ZT12 group at ZT4). Mice had free access to water during the fasting period. The experimental flowchart is illustrated in Fig 1.

### Time series sampling strategy

The NCD, HFD and PAH groups underwent time series RNA sequencing and blood melatonin detection experiments over a 24-hour period. Based on the dynamic sampling protocol established in previous studies [24], the procedure was conducted as follows: Following the intervention, mice from all three groups were euthanized at every 4 Zeitgeber times (ZTs 0, 4, 8, 12, 16, and 20) for sample collection. At each time point, three mice per group were randomly selected and sacrificed, resulting in a total of six sampling time points over a 24-hour period. During the dark cycle, sampling was conducted under dim red light (<5 lux), as described in a previous study [25]. Sampling at each time point was completed within 1 hour, accounting for time differences.

### Body weight measurement and biochemical assays

Body weight of the mice was measured daily at 8:00 PM. Lee’s index (weight^0.33^ * 10^3^/ nasoanal length) [26] is estimated at the end of modelling and intervention. Following intraperitoneal administration of sodium pentobarbital (50 mg/kg), retro-orbital venous plexus puncture was performed to obtain blood samples. Serum lipid profiles, including triglycerides (TG, Cat# 141722013; Mindray), total cholesterol (TC, Cat# 141622017), high-density lipoprotein cholesterol (HDL-C, Cat# 142122016), and low-density lipoprotein cholesterol (LDL-C (Cat# 142022015), were analyzed using an automated biochemistry analyzer (Mindray BS-200). Hepatic function markers aspartate aminotransferase (AST, Cat# 140222011) and alanine aminotransferase (ALT, Cat# 140122010) were concurrently assessed. Additionally, plasma free fatty acid (FFA) concentrations were determined via enzymatic colorimetry (Solarbio, BC0590 assay kit).

### Oral glucose tolerance test (OGTT)

OGTT was conducted as previously described [27]. Following an 8-hour fast, the mice were administered a 2 g/kg dextrose solution by oral gavage. Blood glucose dynamics were monitored at five timepoints (0, 30, 60, 90, 120 min) through tail vein puncture with a Sinocare GA-3 biosensor, and metabolic response was quantified via AUC analysis of the glycemic curve.

### ELISA

Following the detailed steps in the mouse ELISA kit manual, a full-wavelength multifunctional microplate reader was used to measure serum interleukin-6 (IL-6), leptin (Lep) and tumor necrosis factor-alpha (TNF-α) (Enzyme-linked Biotechnology Co., Ltd., NO. ml063159, ml002095, ml002287; Shanghai, China). A time series of blood samples was applied to a mouse melatonin ELISA kit produced by Elabscience Biotechnology Co., Ltd. (Wuhan, China; NO. E-EL-M0788c).

### H&E staining

Following previously established protocols [28], 4-μm-thick liver tissue and 10-μm-thick adipose tissue sections for H&E staining. Then imaged and analyzed via a digital pathology scanning system (Soptop, Ningbo, China).

### Oil Red O staining

To assess differentiation, epididymal adipose tissue (EAT) was stained with Oil Red O. After three cycles of phosphate-buffered saline (PBS) rinsing (5 min each), the specimens underwent fixation with 4% paraformaldehyde (PFA) for 30 min at room temperature. Subsequent PBS washes (×2) preceded staining with Oil Red O (60% saturation) for 10–15 min, followed by a 60% isopropanol rinse to remove excess dye. Then imaged via a digital pathology scanning system (Soptop, Ningbo, China).

### Time series rectal temperature detection

Following the intervention, the rectal temperatures of the mice were measured at every 4 hours (ZTs 0, 4, 8, 12, 16, and 20). Before measurement, the mice were stimulated to defecate. A veterinary thermometer lubricated with glycerin was inserted approximately 1 cm into the rectum. The mice were held still for 30 seconds, and two readings for average as the temperature for that time point. During the nighttime measurements, dim light was maintained below 5 lux. Finally, a rectal temperature curve was generated for each mouse.

### Time series RNA sequencing

The time series of liver samples underwent the RNA-sequencing protocol. Fifty milligrams of liver tissue was homogenized, with three replicates. Total RNA was isolated using TRIzol reagent (Takara, Japan). The quality, purity, and concentration of the extracted RNA were assessed using a spectrophotometer (Nanodrop 2000, Thermo Fisher Scientific, USA). The cDNA library preparation, sequencing, and bioinformatics analysis were performed by OE Biotech Co., Ltd. (Shanghai, China) following standard protocols [29]. The sequencing libraries were processed using the Illumina NovaSeq 6000 platform. The reference genome was indexed with HISAT2, followed by alignment of high-quality paired-end reads. Gene expression levels were quantified in FPKM (fragments per kilobase of exon per million mapped fragments) and converted to read counts for downstream analysis.

### Rhythm analysis and Venn analysis

JTK_CYCLE [30] was applied to detect the rhythm of the gene’s expression at different ZTs in each group. On the basis of the results of JTK_CYCLE parameters such as the amplitude, period, and phase of rhythmic fluctuations were further analyzed. This study uses the JTK version 3 R package for calculations, with a *P* value < 0.05 as the criterion for rhythmic significance. Venn analysis was generated via EVeen (http://www.ehbio.com/Esx) [31].

### Enrichment and upstream regulator analysis

On the basis of the results of gene annotation, the Gene Ontology (GEO, http://www.geneontology.org) database was imported to categorize gene functions into three categories: Cellular Component (CC), Molecular Function (MF), Biological Process (BP). The WEGO online annotation tool (http://wego.genomics.org.cn/cgi-bin/wego/index.pl) was used for GO plotting. The results of the gene analysis were further annotated via the Kyoto Encyclopedia of Genes and Genomes (KEGG) metabolic pathway database [32,33] following the citation guidelines provided at http://www.kegg.jp/kegg/kegg1.html, and a hypergeometric test was employed to analyze the significantly enriched pathways for the genes.

### Quantitative real‑time PCR analysis

Total RNA (1 µg) was reverse transcribed into cDNA. Following Oxford Nanopore Technologies’ protocol, sequencing libraries were prepared and analyzed on the PromethION platform (Biomarker Technology, Beijing). Select genes were validated by qPCR using SYBR Green Master Mix (Vazyme Biotech, Nanjing). Gene-specific primers (Table 1) were designed and synthesized by FOREGENE Biotech (Chengdu). For normalization, target gene expression was calculated relative to β-actin. Relative mRNA expression changes were quantified using the 2^−ΔΔ^CT method.

**Table 1:** Primer sequences for qRT‒PCR.

Gene	Forward primer (5′–3′)	Reverse primer (5′–3′)
*Sphk1*	ACTGATACTCACCGAACGGAA	CCATCACCGGACATGACTGC
*S1pr4*	CCCTGGCCGTGTTCAACTC	ACCGAGAAGTCCGAAAACTGT
*Traf2*	GGGGACCAGGTTAGAAGCC	GGAAAGGCCGAACTACTCTCT
*NF-κB*	ATCCCTACGGAACTGGGCAA	ATCCCCTCTGTTTTGGTTGCT
*β-actin*	CCACCATGTACCCAGGCATT	CAGCTCAGTAACAGTCCGCC

### Western blot analysis

Using a standardized western blot protocol, we quantified the expression of the target protein [34]. The primary antibodies used were as follows: anti-NF-κB (ab16502, Abcam, 1:1000), anti-TRAF2 (A0962, ABclonal, 1:1000), and anti-GAPDH (ab9485, Abcam, 1:3000). The protein bands were photographed and quantified via an E-blot touch imager (E-blot, China).

### Statistical analysis

Analysis and plotting were conducted via SPSS 24.0, GraphPad 9.4, and Origin 2021 software. The measurement data are displayed as the mean ± standard deviation (x ± SD) or (mean ± quartile). Comparisons of two groups via t tests, with Welch’s correction applied for nonnormally distributed data. For comparisons among multiple groups one-way ANOVA were applied, followed by the least significant difference (LSD) test. For skewed distribution samples, the Kruskal‒Wallis nonparametric test was used to obtain the *P* values for intergroup differences, and Dunn’s test was applied for post hoc pairwise comparisons. For intergroup correlations, normally distributed samples were analyzed via Pearson correlation analysis, whereas nonnormally distributed samples were analyzed via Spearman correlation analysis, with *P* < 0.05 considered statistically significant.

## Results

### PA improved HFD-induced obesity

**Fig 2:**
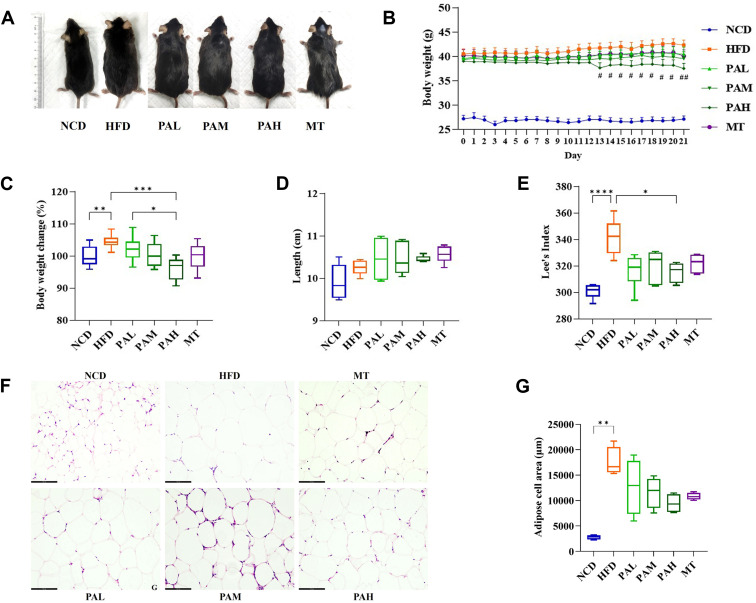
PA improved HFD-induced obesity. **(A)** Representative images of body shape of mice in each group after 21-day intervention. **(B, C)** Effects of PA treatment on body weight (B) and body weight change rate (C) in HFD-induced obese mice. Statistical significance was determined by one-way ANOVA followed by post hoc test (n = 10 per group). **(D, E)** Body length (D) and Lee’s index (E) of mice in different groups. Statistical analysis was performed using the Kruskal–Wallis nonparametric test (n = 6 per group). **(F)** Representative H&E staining images of EAT from mice in different groups. Scale bar: 1 μm. **(G)** Quantification of adipocyte cell area in EAT (n = 4 per group). Statistical analysis was performed using the Kruskal–Wallis nonparametric test. Data are presented as mean ± SEM. ^*^ P < 0.05, ^**^ P < 0.01, ^***^ P < 0.001, ^****^P < 0.0001 versus the NCD group. ^#^ P < 0.05, ^##^ P < 0.01, ^###^ P < 0.001 versus the HFD group.

After 9 weeks of HFD feeding, the mice exhibited significant weight gain and impaired glucose tolerance (S1 Fig). Based on one-way ANOVA, the PAH (high-dose PA) group exhibited significantly lower body weight than the HFD (high fat diet) group on day 13 of the intervention (*P* = 0.014) (Fig 2B). Over the entire 21-day period, the PAH group maintained consistently lower body weight compared with the HFD group (*P* = 0.004) (Fig 2B). Similarly, the MT (melatonin), PAM (medium-dose PA), and PAL (low-dose PA) groups showed attenuated weight gain relative to the HFD group (Fig 2A, B). Body weight decreased significantly in the PA and MT groups, with the most pronounced reduction observed in the PAH group (*P* = 0.003) (Fig 2C). Lee’s index was notably decreased following PA intervention, with the PAH group showing the most significant decrease among all groups (*P* = 0.038) (Fig 2E). No significant differences in body length were observed between the groups (Fig 2D). H&E staining revealed that adipocyte size was significantly reduced in the PA and MT groups (Fig 2F, G). These results indicate that PA intervention effectively reduced body weight and attenuated fat accumulation.

### PA rescued HFD-induced metabolic syndrome

**Fig 3:**
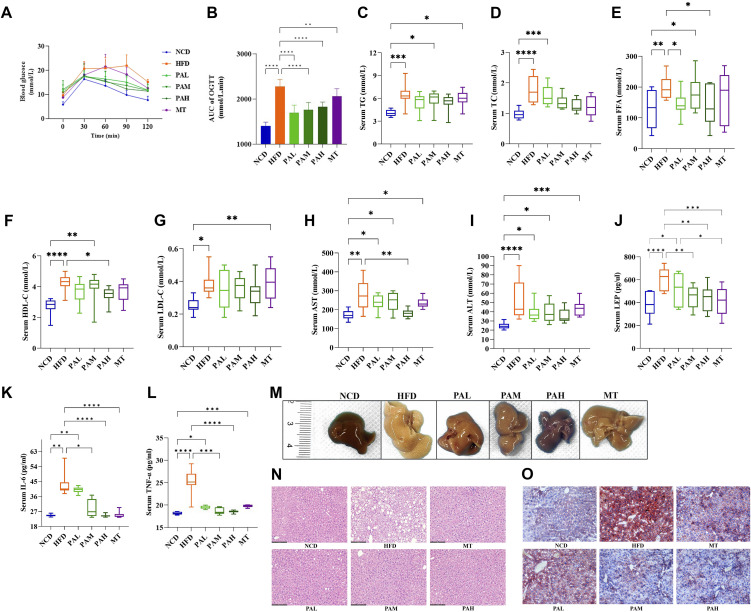
PA rescued HFD-induced metabolic syndrome. PA-induced reductions in blood glucose during the OGTT (A) and area under the curve (AUC) analysis using one-way ANOVA **(B)**. PA decreased serum TG, TC, FFA, HDL-C, LDL-C, AST, and ALT (C–I). PA reversed HFD-induced leptin resistance (J) and reduced serum IL-6 and TNF-α levels **(K & L)**. Representative images of liver morphology (M) and H&E (N) and Oil Red O (O) staining of liver sections from each group. Data are presented as mean ± SEM (n = 10 per group). For panels B–L, statistical significance was determined by one-way ANOVA. ^*^ *P* < 0.05, ^**^ *P* < 0.01, ^***^ *P* < 0.001, ^****^P < 0.0001.

Although food intake displayed no difference on each intervention day, energy intake was notably high in the HFD group (S2 Fig). Whereas, we think that the fluctuation in food intake and energy consumption during the preceding week might be attributed to the intervention administered through intraperitoneal injection. The OGTT was markedly decreased after PA intervention (Fig 3A & B). PA intervention significantly improved the serum TC, TG, FFA, HDL-C, and LDL-C levels compared with HFD group (Fig 3C - G) and the detail results could be found in S1 Table. In addition, the levels of AST and ALT decreased in the PA groups (Fig 3H & I). Obesity is often defined as a state of chronic, low-level body inflammation [35]. The levels of IL-6, TNF-α and leptin decreased in the PA groups (Fig 3J - L). The results were shown in S1 Table. Both the staining results also indicated that PA markedly reduced adipose accumulation in the livers of HFD-fed mice (Fig 3M - O). These findings suggest that PA intervention significantly ameliorates HFD induced glucose and lipid metabolism dysregulation in mice. Additionally, PA administration yielded dose-dependent improvements in glucose homeostasis, lipid metabolism, and hepatic inflammation (Fig 3). Notably, the PAH regimen (10 mg/kg) exerted the most pronounced metabolic benefits across all parameters evaluated. Consequently, this optimal dose was selected for all subsequent chronobiological investigations. Therefore, PAH is the most effective concentration for improving obesity and Metabolic Syndrome (MS); therefore, we chose PAH for subsequent circadian experiments.

### PA restored HFD-induced melatonin and temperature rhythms

**Fig 4:**
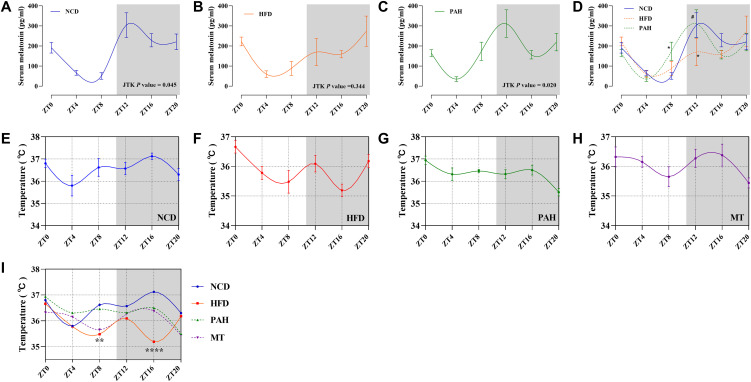
PA repaired HFD-induced changes in melatonin and temperature rhythms. **(A-D)** PAH restored the 24-hour melatonin circadian rhythm in obesity mice (n = 3 in each group). (E–I) PAH rescued the temperature rhythm in obesity mice (n = 5 per group). Data are presented as mean ± SEM with individual data points plotted to illustrate effect sizes and distribution. Statistical significance was evaluated using two-way ANOVA for (D) and **(I)**. In panel **(D)**, hashtags (#, ##, ###) indicate significant differences compared with the HFD group (# P < 0.05, ## P < 0.01, ### P < 0.001). In panel **(I)**, asterisks (*, **, **) indicate significant differences compared with the NCD group (* P < 0.05, ** P < 0.01, *** P < 0.001).

Time series analysis of the serum melatonin levels revealed the HFD group lacked rhythmicity (*P* = 0.344), while the serum melatonin in PAH intervention group display rhythmicity (*P* = 0.020) (Fig 4A - D). Besides, the mice in all the groups presented the lowest serum melatonin levels between ZT 4 and ZT 8, equivalent to 12:00 AM to 4:00 PM. While the PAH and NCD groups presented peak melatonin levels between ZT 12 and ZT 16 (8:00 PM to 12:00 AM), the peak was delayed significantly in the HFD. Moreover, the HFD exhibited a significant reduction in melatonin secretion at ZT 12 (*P* < 0.05) (Fig 4A - D). PAH intervention effectively rescued the disrupted rectal temperature rhythm in HFD-fed mice, as shown by 24-hour dynamic temperature measurements (Fig 4F & G). Specifically, PAHs restored temperatures at ZT 8 and ZT 16 to levels comparable with those of the NCD group (Fig 4E & I). Similarly, MT increased the temperature at ZT 16, but the difference from NCD was not significant. At ZT 8, the trend in the MT group resembled that in the NCD group, suggesting that both PAH and melatonin can effectively restore HFD-induced circadian temperature disturbances (Fig 4H).

### PA restored the liver gene oscillation rhythm in HFD-induced obese mice in the Sphingolipid pathway

**Fig 5:**
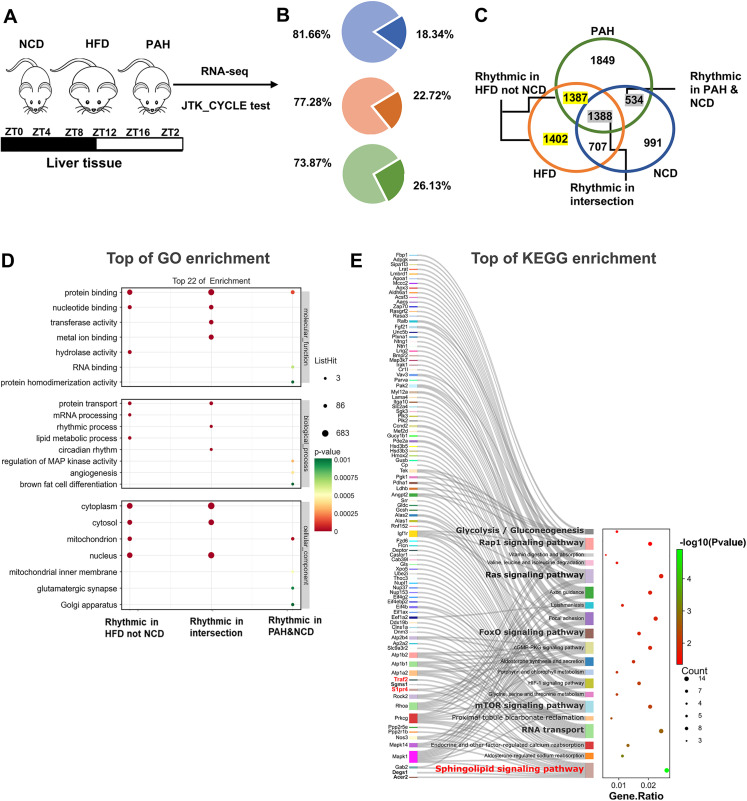
PA restored the liver gene oscillation rhythm of HFD-induced obese mice in the Sphingolipid pathway. The general steps of a time series RNA sequencing study **(A)**. Rhythmic gene rates in the NCD, PAH and HFD groups **(B)**. A total of 534 genes whose rhythmic expression was rescued by PA were analyzed by Venn **(C)**. GO analysis of the PA rescue rhythmic genes **(D)**. KEGG analysis of the PA rescue rhythmic genes **(E)**. The sample size was n = 18 per group, with n = 3 at each time point within each group.

Time series RNA-seq of the liver which collected at every 4 ZT time point was performed, and rhythmicity was quantified via the JTK_CYCLE test (Fig 5A). The results revealed 3,620 (18.34%) rhythmic genes in the NCD group, 4,884 (22.72%) in the HFD group, and 5,158 (26.13%) in the PAH group (Fig 5B). Among the clock-related genes analyzed, we found that the core clock regulators selected for this study—which represent the most widely studied and critical components of the machinery—maintained consistent rhythmic stability. However, the *Clock* and *Id2* genes showed variations in rhythmicity in HFD group (Table 2). Venn analysis revealed that a HFD caused 2,789 genes to become rhythmic and 1,525 genes to lose rhythmicity. In contrast, PAH intervention restored rhythmicity to 534 genes disrupted by HFD (Fig 5C). These results indicate that the core circadian gene was relatively stable in the liver, whereas oscillating liver genes are disrupted by a HFD and can be partly rescued by PAH. To investigate the molecular mechanisms by which PA modulates HFD-induced obesity and circadian rhythm disruptions, we conducted GO and KEGG pathway enrichment analyses based on the 534 genes. The Sphingolipid pathway was the most significantly enriched, and this pathway is related to the differentiation of adipose and the accumulation of lipids, which contribute to the pathogenesis of obesity [36] (Fig 5D - E).

**Table 2:** Rhythm parameters of the liver core circadian clock gene.

Group	Gene	*P* value	Period	Phase	Amplitude
HFD	*Arntl*	<0.0001^****^	24	22	0.67
	*Bhlhe40*	0.0001^***^	24	14	12.06
	*Bhlhe41*	0.0002^***^	24	8	0.20
	*Clock*	0.0623	20	2	0.76
	*Cry1*	<0.0001^****^	24	18	4.13
	*Cry2*	0.0003^***^	24	12	2.74
	*Dbp*	<0.0001^****^	24	10	19.95
	*Id2*	1.0000	20	0	40.25
	*Nr1d1*	＜0.0001^****^	24	8	12.87
	*Nr1d2*	<0.0001^****^	24	10	8.57
	*Per1*	0.0017^**^	24	14	1.31
	*Per2*	<0.0001^****^	24	12	2.34
NCD	*Arntl*	<0.0001^****^	20	2	0.47
	*Bhlhe40*	0.1041	24	14	18.46
	*Bhlhe41*	0.0001^***^	20	10	0.31
	*clock*	0.0075^**^	24	0	1.15
	*Cry1*	<0.0001^****^	24	20	3.84
	*Cry2*	0.0008^***^	20	10	2.13
	*Dbp*	<0.0001^****^	20	10	32.90
	*Id2*	0.0075^**^	20	4	12.40
	*Nr1d1*	<0.0001^****^	24	6	11.05
	*Nr1d2*	<0.0001^****^	24	10	8.85
	*Per1*	0.0359^*^	20	14	0.66
	*Per2*	<0.0001^****^	24	14	3.11
PAH	*Arntl*	<0.0001^****^	24	22	0.522529
	*Bhlhe40*	<0.0001^****^	20	10	11.96403
	*Bhlhe41*	<0.0001^****^	24	10	0.226774
	*clock*	0.0269	20	4	0.138073
	*Cry1*	0.0003	24	18	2.724114
	*Cry2*	0.0003	20	10	1.464241
	*Dbp*	<0.0001^****^	20	10	23.77536
	*Id2*	0.0199	20	4	0.177193
	*Nr1d1*	<0.0001^****^	24	6	16.09168
	*Nr1d2*	<0.0001^****^	24	10	9.225073
	*Per1*	0.0053	20	14	0.717278
	*Per2*	<0.0001^****^	24	12	2.296596

JTK test p value: * *p* < 0.05, ** *P* < 0.01, *** *P* < 0.001, **** *p* < 0.0001.

### PA modulated the S1PR4/TRAF2 signaling rhythm in the Sphingolipid pathway to alleviate liver inflammation

Focusing on Sphingolipid pathway, we identified *S1pr4* and *Traf2* as hub genes whose rhythmic expression was restored upon PAH intervention. These findings suggest that PAH may restore rhythmicity in the *Sphk1*/S1pr4*/Traf2* pathway, potentially mitigating inflammation in the liver tissue of obese mice.

**Fig 6:**
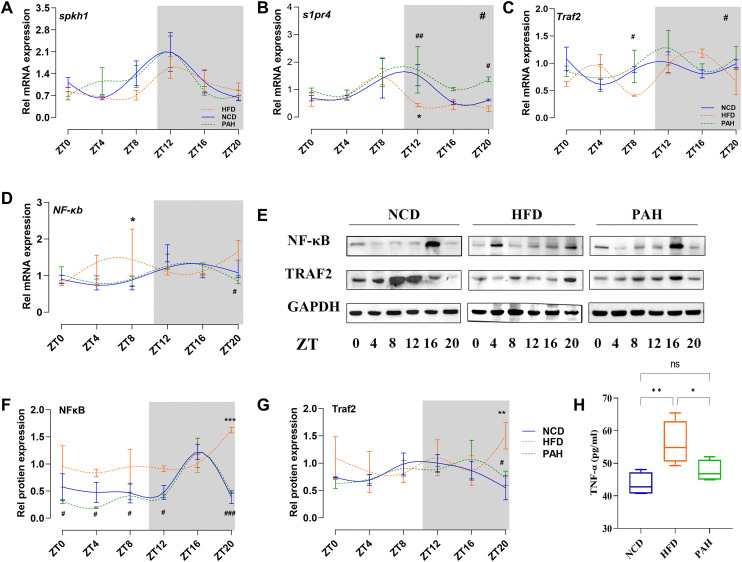
PA modulated the expression of genes and proteins in the Sphingolipid pathway. The circadian expression of genes *sphk1, s1pr4, Traf2, and NF-*κ*B* **(A-D)**. The circadian levels of proteins TRAF2 and NF-κB **(E-G)**. Statistical significance was assessed using two-way ANOVA. The level of TNF-α in liver tissue at the ZT20 time point **(H)**. n = 3 in each group. Statistical significance was determined using one-way ANOVA. Hashtags #, ##, and ### indicate significant differences compared with the HFD group (*P* < 0.05, < 0.01, and <0.001); asterisks *, **, and *** indicate significant differences compared with the NCD group (*P* < 0.05, < 0.01, and <0.001).

The 24-h analysis of the mRNA expression of genes *sphk1, s1pr4, Traf2, and NF-*κ*B* revealed four pieces of information. The dynamic expression results of *sphk1* mRNA revealed that all three groups peaked at ZT12, with no statistically significant differences at any other time point (*P* > 0.05). The mRNA expression of *S1pr4* peaked at ZT12 in both the PAH and NCD groups, whereas it was significantly lower in the HFD group (*P* < 0.05). The overall trend of *Traf2* mRNA expression in the HFD was the opposite of that in the NCD and PAH groups. The *NF-κB* mRNA did not differ between the NCD and PAH groups, and the trend was consistent, with peaks occurring between ZT12 and ZT16, whereas the HFD peaked at ZT8. The expression level in the HFD group at ZT8 was significantly higher (*P* < 0.05), and the PAH level at ZT20 was significantly lower than that in the HFD group (*P* < 0.05). The JTK_CYCLE results indicated that *s1pr4* and *Traf2* exhibited rhythmicity in the NCD and PAH groups (*P* < 0.05), whereas no rhythmicity was detected in the HFD group (*P* > 0.05) (Fig 6A - D). Furthermore, *s1pr4* and *Traf2* are key to the NF-κB pathway. After PAH intervention, NF-κB protein expression in the liver tissue at the six time points was consistent with that of the NCD-fed mice, with significantly high expression at ZT16. The HFD, however, was relatively greater at ZT20 (*P* < 0.05). Additionally, the protein expression levels in the PAH and NCD groups were significantly lower than those in the HFD groups, except at ZT16 (*P* < 0.05). At ZT20, Traf2 protein expression in the HFD group was significantly greater than that in the NCD and PAH groups (*P* < 0.05). There was no difference between the PAH and NCD groups at ZT20 (*P* > 0.05). The proteins NF-κB and Traf2 did not significantly change in any of the three groups (Fig 6E - G).

Traf2 and NF-κB are associated with inflammation regulation, so we detected TNF-α expression in the liver [37]. The TNF-α expression level in the liver tissue homogenate at ZT20 was measured, and the results revealed that the expression level in the HFD group was significantly greater than that in the NCD and PAH groups The results showed that TNF-α levels in the HFD group (54.82 ± 6.781 pg/ml) were significantly higher than those in the NCD group (42.74 ± 3.506 pg/ml; *P* = 0.0051) and the PAH group (46.74 ± 3.360 pg/ml; *P* = 0.0347) (Fig 6H). The results of RT‒qPCR, WB and ELISA indicated that PA may rescue the *s1pr4/Traf2* pathway and regulate the Traf2 and NF-κB proteins to decrease the inflammatory expression of TNF-α in liver tissue.

## Discussion

A HFD can significantly disrupt circadian rhythms, the body’s internal clock, and promote adipose tissue accumulation, potentially contributing to metabolic issues such as obesity and insulin resistance [38]. Our findings indicate that the mechanism of the effects of PA involves improvements in HFD-induced obesity-related metabolic disorders, insulin-related leptin resistance, circadian misalignment and liver inflammation.

PA is naturally found in the wood-rotting fungus *Poria cocos* and is a key component in many foods and traditional Chinese medicines derived from this fungus. It is well known as “Fu-ling” in traditional Chinese medicine, making it a prevalent natural ingredient in various *Poria cocos*-based products [39]. It has many potential health benefits, including anti-inflammatory [40,41], anticancer [42], and antihyperglycemic properties [17]. Notably, PA primarily functions to enhance lipid metabolism by activating the SIRT6 signaling pathway. This activation promotes fatty acid β-oxidation, thereby alleviating hepatic lipid accumulation and potentially aiding in the management of conditions such as Non-Alcoholic Fatty Liver Disease (NAFLD) and obesity [20]. Our results are consistent with previous findings that *Poria cocos and its* based products alleviated HFD-induced obesity, inhibited the accumulation of lipid and regulated glucolipid metabolism [43]. Obesity, considered a complex metabolic disorder, is strongly linked to an increased risk of developing numerous diseases [44]. Our results identify PA as a promising candidate against obesity.

HFD-induced obesity and metabolic syndrome are closely involved in circadian disorders [45,46]. The circadian rhythm, generated by the endogenous biological clock, operates on a 24-hour cycle and regulates various physiological functions such as body temperature, hormone secretion, and energy metabolism [47]. A HFD is often considered a dietary factor that can disrupt the normal circadian rhythm [8], meaning that it can significantly interfere with the body’s natural internal clock, leading to irregular patterns in sleep, eating, and other bodily functions throughout the day. Natural products, including berberine and nobiletin, demonstrate potential in lipid lowering and circadian rhythm restoration, highlighting their promise as therapeutic candidates for obesity treatment [48–50]. PA has a sedative-hypnotic effect, potentially by acting on increasing α- and β-subunits protein levels, but decreased γ-subunit protein levels in GABAA receptors, which are closely associated with sleep and have been demonstrated to prolong sleep in mice [51]. Sleep and diet are both important regulators of biological rhythms [52].

In addition, both melatonin secretion and body temperature are driven by the clock primarily by the suprachiasmatic nucleus (SCN) sited in the hypothalamus, defined as the central clock and therefore reflects our internal circadian rhythm [53,54]. Our results showed that PA could ameliorate HFD-induced changes in melatonin and temperature. Therefore, we hypothesized that PA may also influence biological rhythms. The liver clock regulates various metabolic functions throughout the day, aligning its activity with the body’s natural day‒night cycle [55]. It primarily controls processes such as nutrient uptake, processing, detoxification, and energy homeostasis on the basis of the time of day [56].

Time series RNA-seq data-based bioinformatics analysis is currently one of the main tools used to elucidate the complex molecular mechanisms underlying circadian rhythm disorders [57,58]. It is noteworthy that PA intervention did not simply replicate the rhythmic landscape of NCD mice. While a subset of 1388 genes exhibited conserved rhythmicity across NCD and PAH groups, we also observed genes unique to either condition (991 in NCD-only and 1849 in PAH-only). This discrepancy suggests that PA does not merely “copy” the physiological rhythm of healthy mice, but rather induces an adaptive rhythmic reprogramming to rebuild metabolic homeostasis under post-HFD conditions. Crucially, we focused on the 534 “rescued” genes that lost rhythmicity under HFD but were restored by PA. These genes were significantly enriched in the sphingolipid signaling pathway and lipid metabolism (Fig 5D-E), validating our conclusion that PA ameliorates metabolic dysfunction by reinstating the circadian alignment of core metabolic oscillators, rather than globally resetting all rhythmic genes. As predicted, the enriched signaling pathway is the sphingomyelin pathway, particularly the *S1PR4/TRAF2* signaling pathway, which performs anti-inflammatory functions in the liver. An abnormal sphingomyelin pathway can affect inflammation and insulin production, which can lead to abnormal glucose metabolism and promote the occurrence and development of obesity [59,60]. In addition, mice with whole-body or tissue-specific disruption of the circadian clock genes exhibit higher rate of chronic inflammation, metabolic diseases and obesity [61,62] Our study further revealed that PA could rescue the *S1PR4/TRAF2* signaling rhythm and reduce the NF-κB and TNF-α expression in liver to improve the chronic inflammatory state of HFD-fed mice.

However, this study has several limitations. Although we employed a mouse model, which shares approximately 99% genetic similarity with humans, the remaining 1% disparity may still lead to translational discrepancies. Therefore, further validation in human models is necessary. The number of circadian rhythmic genes identified under HFD conditions in our study differs from those reported in foundational literature such as Eckel-Mahan et al. [63]. This discrepancy likely arises from differences in experimental parameters, including diet formulation, duration of metabolic challenge, liver sampling frequency across Zeitgeber times, and the specific statistical thresholds employed. While recent methodological advances such as the DryR package [64] offer robust direct modeling of RNA-seq count data and direct cross-group rhythmicity comparison, our current analysis utilized JTK_CYCLE with stringent adjusted P-value < 0.05 and amplitude filtering to conservatively control for potential overestimation of rhythmicity. This conservative pipeline ensured high-confidence identification of core circadian regulators relevant to our biological model. Future investigations will directly benchmark JTK_CYCLE against DryR to further refine circadian transcriptomic profiling in diet-induced metabolic disease contexts. Additionally, we did not incorporate a CRISPR-Cas9 experiment to validate the rhythm of *S1PR4/TRAF2* signaling, as time series sampling necessitated many mice. Furthermore, owing to the time series sampling, we sacrificed only three samples in each group at each time point for RNA sequencing. While this sample size is statistically sufficient, it remains relatively small. Further studies were warranted to determine whether modulating the circadian clock with PA represents a viable strategy for reducing inflammation in obese patients.

## Conclusion

In summary, our research elucidates the role of PA function of lipid metabolism through the restoration of circadian rhythm alignment in long-term HFD-fed mice by partially reinstating the rhythmic oscillation of core genes and proteins in the sphingomyelin pathway, alongside inducing adaptive rhythmic reprogramming. This restoration of key metabolic rhythms, combined with the amelioration of chronic inflammation, highlights PA’s potential as a chronotherapeutic agent for obesity and metabolic syndrome. Nevertheless, further investigations are essential to fully decipher comprehend the underlying mechanisms and translate these findings into clinical practice.

### Statement of human and animal rights

All procedures in this study were conducted in accordance with the Institutional Animal Care and Use of Chengdu University of Traditional Chinese Medicine, China and approved by the Committee Chengdu University of Traditional Chinese Medicine laboratory Animal Welfare Ethics Committee (No. 2024107) Chengdu Sichuan, China.

## Supporting information

S1 Fig Obesity was modeled via 9-week HFD feeding. Representative images of body shape (A). HFD feeding contributed to weight gain (B). HFD feeding displayed abnormal glucose tolerance (C & D). n = 10 in each group. * *p* < 0.05, ** *p* < 0.001 and *** *p* < 0.0001.
(TIF)

S2 Fig Food intake and energy intake on intervention days. (TIF)

S1 Table Results of the biochemical analysis of ALT, AST, IL-6, TNF-α, and leptin. n = 10 in each group. asterisks *, **, and *** in the table indicate significant differences compared with the NCD group (p < 0.05, < 0.01, and <0.001). Hashtags #, ##, and ### in the table indicate significant differences compared with the HFD group (p < 0.05, < 0.01, and <0.001).
(DOCX)

S1 File Raw data. This is the raw data of this study.
(XLS)

S2 File FPKM data of the RNA-seq. (XLSX)

S3 File Images for Blot. (PDF)
