# Supplementary figures and images for: Pachymic acid alleviates circadian rhythm disorders in high-fat diet-induced obesity mice via the sphingolipid pathway
Source: PLoS One. 2026 Jul 1;21(7):e0352604. doi: 10.1371/journal.pone.0352604 (PMC13322541; doi:10.1371/journal.pone.0352604)

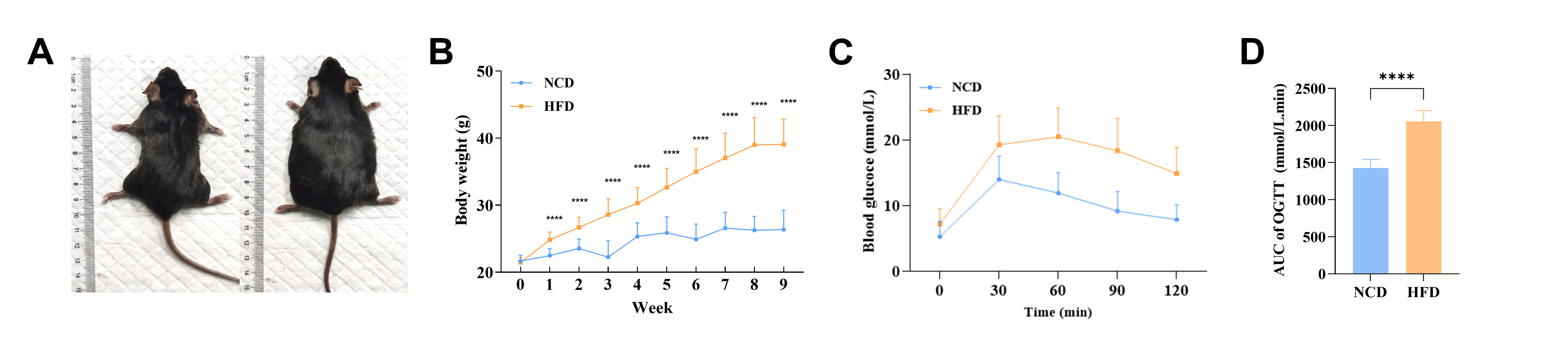

Supplement: S1 Fig — Representative images of body shape (A). HFD feeding contributed to weight gain (B). HFD feeding displayed abnormal glucose tolerance (C & D). n = 10 in each group. * p < 0.05, ** p < 0.001 and *** p < 0.0001. (TIF) [file pone.0352604.s001.tif]

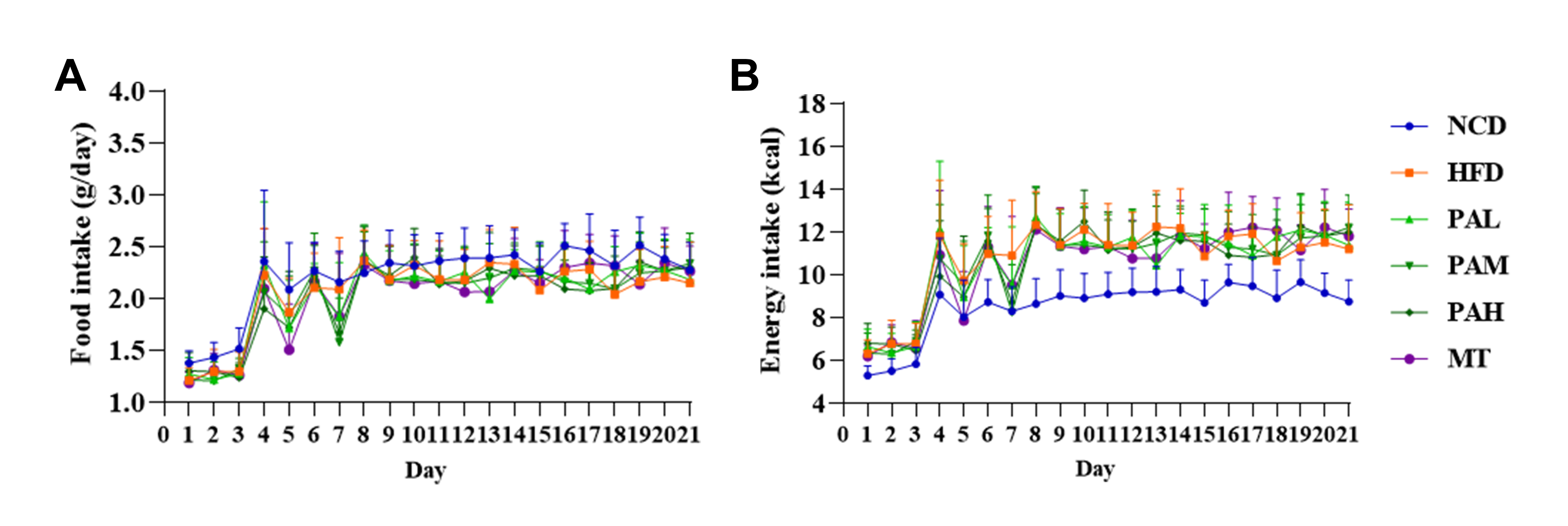

Supplement: S2 Fig — (TIF) [file pone.0352604.s002.tif]
