# Supplementary material for: Pachymic acid alleviates circadian rhythm disorders in high-fat diet-induced obesity mice via the sphingolipid pathway
Source: PLoS One. 2026 Jul 1;21(7):e0352604. doi: 10.1371/journal.pone.0352604 (PMC13322541; doi:10.1371/journal.pone.0352604)
Supplement: S1 Table — n = 10 in each group. asterisks *, **, and *** in the table indicate significant differences compared with the NCD group (p < 0.05, < 0.01, and <0.001). Hashtags #, ##, and ### in the table indicate significant differences compared with the HFD group (p < 0.05, < 0.01, and <0.001). (DOCX) [file pone.0352604.s003.docx]

Table S1: Results of the biochemical analysis of ALT, AST, IL-6, TNF-α, and leptin.

| **Group** | **HFD** | **PAL** | **PAM** | **PAH** | **MT** | ***F*** | ***P*** |
| --- | --- | --- | --- | --- | --- | --- | --- |
| **TG** | 1.695±1.355^****^ | 1.490±1.300^***^ | 1.315±1.170 | 1.170±1.090 | 1.195±0.940 | 8.938 | <0.0001 |
| **TC** | 6.325±5.96^***^ | 5.830±4.670 | 6.185±5.325^*^ | 5.675±5.180 | 6.020±5.473^*^ | 5.848 | 0.0002 |
| **FFA** | 191.2±165.3^**^ | 139.3±126.7^#^ | 174.1±130.1^*^ | 129.1±87.4^#^ | 189.8±73.1 | 2.182 | 0.0696 |
| **HDL-C** | 4.320±4.025^****^ | 3.835±3.160 | 4.160±3.873^**^ | 3.560±3.255^##^ | 3.915±3.145 | 6.461 | <0.0001 |
| **LDL-C** | 0.360±0.338^*^ | 0.345±0.240 | 0.375±0.298 | 0.340±0.268 | 0.395±0.295^**^ | 3.529 | 0.0078 |
| **ALT** | 42.75±36.33^****^ | 36.45±32.05^*^ | 37.15±30.30^*^ | 32.20±30.23 | 43.70±35.95^*^ | 6.027 | 0.0002 |
| **AST** | 272.4±212.3^**^ | 239.5±214.0^*^ | 252.4±199.8^*^ | 181.2±162.3^##^ | 229.6±217.1^*^ | 7.952 | <0.0001 |
| **IL-6** | 40.47±39.66^**^ | 40.23±38.69^**^ | 26.80±24.29^##^ | 24.38±23.99^####^ | 24.18±23.779^####^ | 59.03 | <0.0001 |
| **TNF-α** | 25.15±24.47^****^ | 19.44±19.25^*^ | 18.42±18.139^###^ | 18.60±18.459^####^ | 19.90±19.61^***^ | 58.08 | <0.0001 |
| **LEP** | 627.4±498.8^****^ | 534.7±356.9^*^ | 467.6±359.9^##^ | 452.3±325.2^##^ | 421.8±302.5^###^ | 5.005 | 0.0008 |

n=10 in each group. asterisks *, **, and *** in the table indicate significant differences compared with the NCD group (*p* < 0.05, <0.01, and <0.001). Hashtags #, ##, and ### in the table indicate significant differences compared with the HFD group (*p* < 0.05, <0.01, and <0.001).
