# Supplementary material for: Pachymic acid alleviates circadian rhythm disorders in high-fat diet-induced obesity mice via the sphingolipid pathway
Source: PLoS One. 2026 Jul 1;21(7):e0352604. doi: 10.1371/journal.pone.0352604 (PMC13322541; doi:10.1371/journal.pone.0352604)
Supplement: S3 File — (PDF) [file pone.0352604.s006.pdf]

Images for Blots

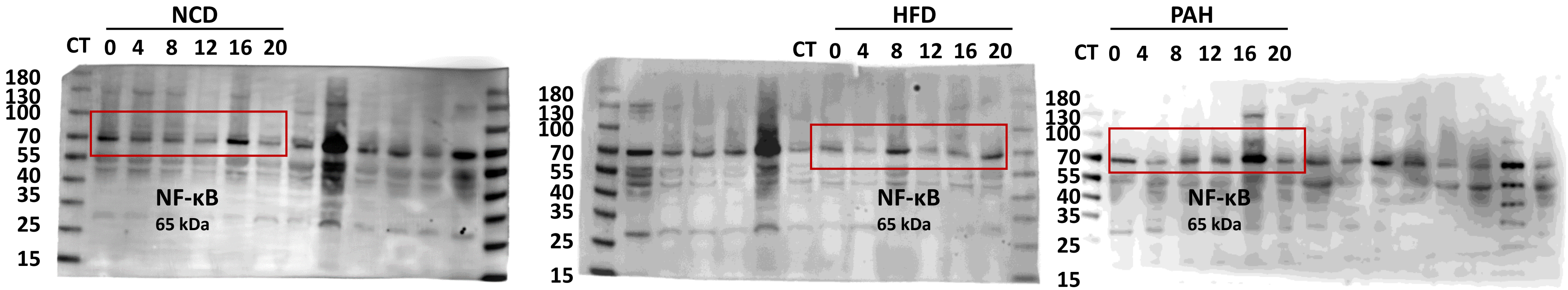

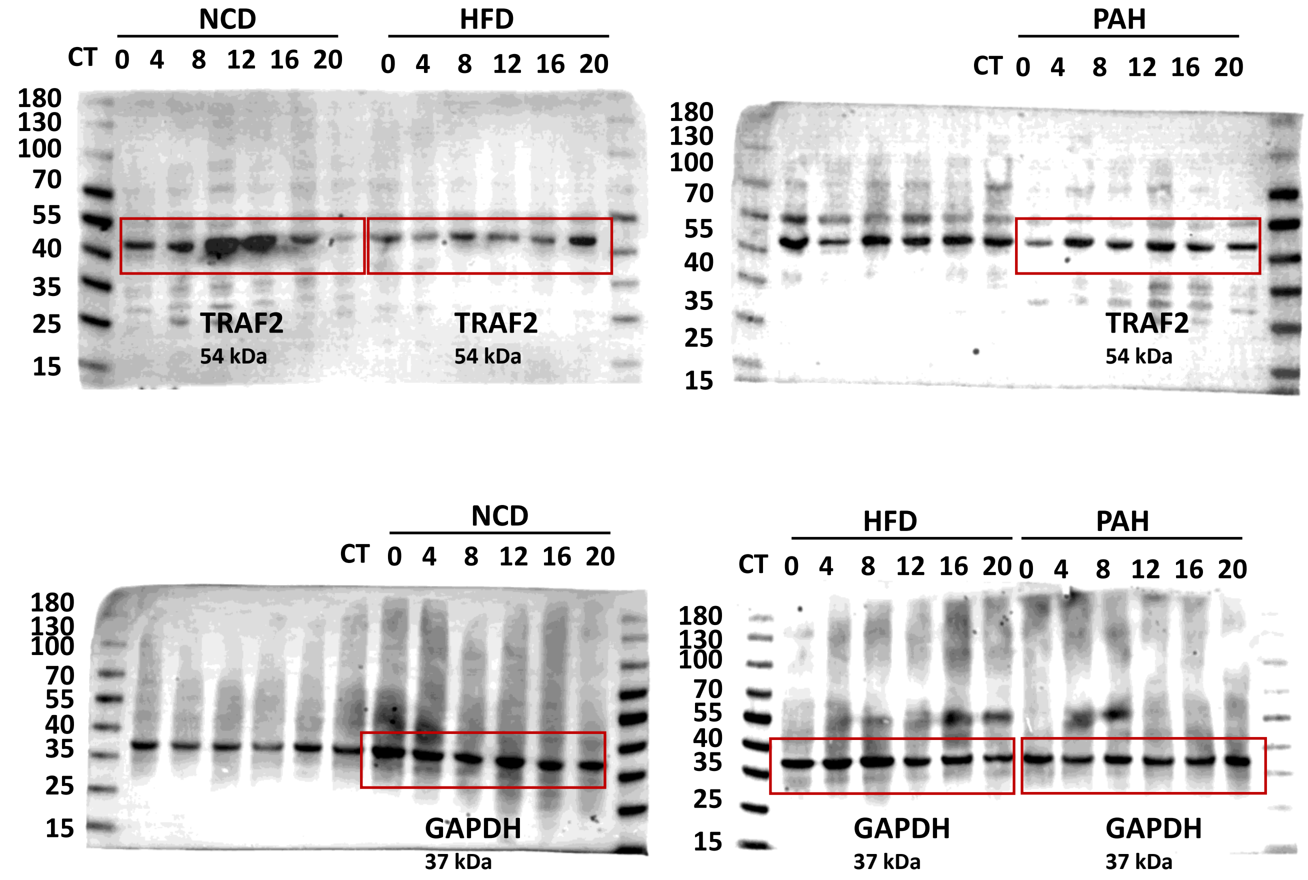

Figure of blots: All images presented are uncropped original blots. The red boxes highlight the bands included in the analysis, while unmarked bands were excluded. Abbreviations: NCD, normal control diet; HFD, high-fat diet; PAH, PA High; ZT, circadian time. The numbers (0, 4, 8, 12, 16, 20) on each lane indicate the respective time points within each experimental group. The molecular weights (kDa) of the target proteins are labeled on the blots.
